# Unmet Needs and Service Priorities for ADHD in Australia: AI-Assisted Analysis of Senate Inquiry Submissions

**DOI:** 10.3390/ijerph23010123

**Published:** 2026-01-19

**Authors:** Blair Hudson, Sam Connell, Anie Kurumlian, Anjali Fernandes, Habib Bhurawala, Alison Poulton

**Affiliations:** 1 Deployscience Labs, Sydney, NSW 2000, Australia; blair@deployscience.com; 2 School of Medicine, University of Notre Dame Australia, Sydney, NSW 2007, Australia; sam.connell@my.nd.edu.au (S.C.); anie.kurumlian@my.nd.edu.au (A.K.); anjali.fernandes@my.nd.edu.au (A.F.); habib.bhurawala@health.nsw.gov.au (H.B.); 3 Nepean Clinical School, The University of Sydney, Sydney, NSW 2050, Australia; 4 Nepean Hospital Penrith, Nepean Blue Mountains Local Health District, Kingswood, NSW 2747, Australia

**Keywords:** attention-deficit hyperactivity disorder (ADHD), Adult ADHD, large language model, AI assisted qualitative analysis, psychiatric services

## Abstract

Objective: To analyse written submissions from individuals and families with lived experience of attention-deficit hyperactivity disorder (ADHD) to the 2023 Australian Senate Inquiry, using artificial intelligence (AI)-assisted thematic analysis. The aim was to identify priority concerns, service needs, and community-proposed solutions. Methods: A mixed-methods study of 505 publicly available submissions from individuals with ADHD and their families. Submissions were analysed using large language model (LLM)-assisted data extraction and thematic clustering, with human validation and review. Main Outcome Measures: Frequency and thematic distribution of (1) problems experienced; (2) services wanted; and (3) solutions suggested. Results: Thematic analysis of 480 eligible submissions revealed high costs and long wait times for assessment and treatment (each cited by 46%), lack of specialised care (39%), diagnostic delays (36%), and gender bias (27%). The most common service request was for affordable and accessible ADHD-specific care (71%), followed by services tailored to diverse populations and life stages. Proposed solutions focused on Medicare-funded access to psychological and psychiatric services (68%), expanded roles for general practitioners, improved provider training (39%), and recognition of ADHD under the National Disability Insurance Scheme. Submissions also highlighted misalignment between current clinical guidelines and public expectations. Conclusions: The findings highlight substantial unmet needs and systemic barriers in ADHD diagnosis and care in Australia. The AI-assisted analysis of consumer submissions offers a scalable method for integrating lived experience into policy development, providing numerical weighting to the individuals’ responses. Coordinated reforms in access, funding, and workforce training are needed to align services with both clinical evidence and community expectations.

## 1. Introduction

Attention-deficit hyperactivity disorder (ADHD) is a neurodevelopmental disorder defined by impaired levels of inattention, organisation, and/or hyperactivity-impulsivity [1]. Although previously considered a disorder of childhood, it is increasingly recognised that functional impairment persists into adulthood in around 60–86% of individuals diagnosed [2].

Untreated ADHD has been associated with poorer outcomes across a range of diverse health and social domains [3,4,5]. The prevalence of ADHD in Australia is estimated at 6–10% in children and adolescents and 2–6% in adults. This equates to around 800,000 Australians and an estimated annual cost of approximately AUD 20 billion [6].

The prevalence of ADHD in Australia, based on prescribing data, has increased two-fold from 2013 to 2020 [7]. International data show comparable trends in America [8] and Finland [9].

The surge in ADHD diagnoses may be attributed to heightened awareness of ADHD, particularly among adults and females, two groups historically underdiagnosed [10]. Additionally, during the COVID-19 pandemic, the loss of the structural and social supports consequent to working from home may have exacerbated symptoms of ADHD and contributed to higher diagnosis rates [11].

The capacity of support services has been inadequate to meet this demand. In 2003 the Western Australian (WA) Parliament conducted an inquiry into ADHD [12]. This was in response to a disproportionately high use of stimulant treatment in WA. A consistent theme among the 83 submissions was lack of other available treatment options, these being expensive or in short supply. There was also frustration that little had been carried out to address the shortage in multidisciplinary assessment and treatment, despite the recommendations in previous reports. Twenty years later the WA Green Party senator Jordon Steele-John was instrumental in advocating for a federal inquiry. In 2023, the Australian Senate duly conducted an inquiry into the barriers to consistent, timely, and best practice assessment of attention deficit hyperactivity disorder (ADHD) and support services for people with ADHD [13].

To date, the ADHD community has not seen any commitment by the federal government that is in any way comparable to the National Roadmap to Improve the Health and Mental Health of Autistic People [14], which followed the Senate Inquiry into services, support, and life outcomes for autistic Australians [15]. The one recommendation from the present inquiry that was supported by the government was for uniform Australia-wide prescribing rules for ADHD medications [16]. However, this would require co-operation between the different jurisdictions, each state and territory having its own legislation around stimulant prescribing.

Artificial intelligence (AI) is increasingly being incorporated into the analysis of health data and has even been proposed as a medical device for legislative purposes [17]. The main applications relate to analysis of patient records and laboratory data such as pathology and radiology reports. Such data can be used to train computers to correlate images with their reports (supervised learning) or to use the data to identify new relationships (deep learning), which may assist with diagnosis, or trends that can be used to predict and improve patient flow (predictive analysis and data-driven intelligence). The Health Data Semantics and Interoperability group uses natural language processing (NLP) to ‘process medical narratives such as pathology reports or medical literature’ [17]. Natural language requires processing before it can be analysed appropriately by AI. Progress in this area using vast troves of internet-based data has enabled the development of large language models (LLMs) that can be used to analyse text. By using NLP, AI can enhance the efficiency of qualitative health research [18,19]. However, AI can lead to misleading data interpretation and requires quality control by human moderators [20].

The purpose of a parliamentary inquiry is to gather evidence and provide recommendations to government, with a view to policy improvement. Such inquiries generate large amounts of qualitative data from stakeholders that is usually analysed narratively. An alternative approach is to regard parliamentary inquiries as a ‘natural experiment’ that can be subjected to systematic analysis. AI has been used for this purpose in other settings. These demonstrate that systematic coding can be applied at scale to large datasets of qualitative data and might in principle identify dominant themes such as stakeholder priorities and gaps in services, indicating its potential value for ADHD service design. However, the only example we could find of an LLM being used to analyse submissions to a parliamentary inquiry involved an experiment conducted by the NSW Productivity and Equality Commission in 2024 [21]. They used a trained LLM to assess the quality of summaries of submissions prepared by LLMs against those prepared by humans. They found that the LLM was less good for capturing the complexity and nuance, which was seen as a limitation to the use of AI for this purpose.

The aim of this AI analysis was to complement and add weight to the recommendations of the official report of the Senate Inquiry [13] by adding numerical data documenting the magnitude of problems experienced, together with the level of support for the different services and potential solutions from those with lived experience of ADHD. The findings may be used to prioritise service reform that addresses existing gaps in ADHD care and support.

## 2. Materials and Methods

### 2.1. Study Design

A mixed-methods approach was employed, combining artificial intelligence (AI) and natural language processing (NLP) techniques with traditional quantitative analysis to extract and categorise data. We used the OpenAI model gpt-4o-mini (OpenAI, San Francisco, CA, USA) for structured extraction, validation, clustering label generation, and summarisation steps. The submissions were the only data provided, together with explicit prompts for data extraction. Importantly, the Senate Inquiry terms of reference and the final report were not provided to the model during extraction or clustering.

### 2.2. Data Collection/Extraction

**Figure 1:**
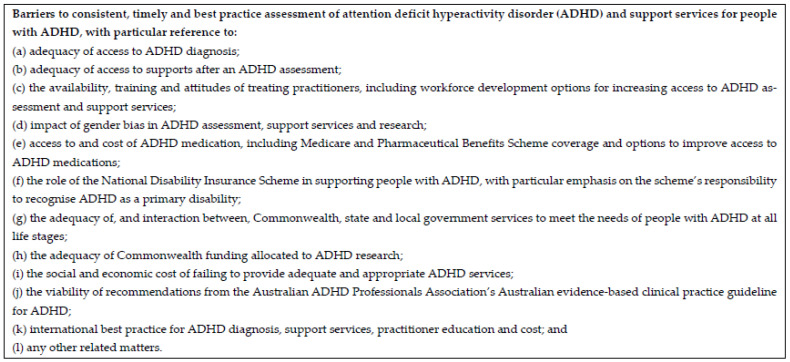
Senate Inquiry Terms of Reference for public submissions [13]. https://www.aph.gov.au/Parliamentary_Business/Committees/Senate/Community_Affairs/ADHD/Terms_of_Reference, accessed on 24 November 2025.

Between March and June 2023, the Australian Parliament invited submissions to the Senate Inquiry via the website of the Senate Community Affairs Reference Committee, giving 12 terms of reference [13] (Figure 1). Submissions were also invited from organisations, key stakeholders, and individuals. The committee received 701 submissions and held three public hearings (Canberra, 29 June 2023; Perth, 24 July 2023; Melbourne, 26 September 2023). The submission process required written responses and access to the public call. The submissions were made freely available on the committee website [13]. Our study was filtered to include only the 505 written submissions from individuals with lived experience of ADHD.

### 2.3. Data Validation

A structured large language model (LLM) prompt was developed and aligned to three key questions: (1) What are the current problems experienced by individuals with ADHD? (2) What services do they want? (3) What solutions do they suggest? The LLM was validated using three human annotators. This first involved a learning sample of 5 submissions. The LLM was used to extract information, organised into the three categories: ‘current problems,’ ‘solutions suggested,’ and ‘services wanted.’ Any available demographic data (respondent gender, state of residence, age, parental status, and relationship to the person with ADHD) were also extracted. These five learning examples were presented to the human annotators to demonstrate the structure to be followed for the individual data extraction.

To avoid additional bias the three human annotators were blinded to the Senate Inquiry terms of reference, official report, and government response. Each annotator independently extracted information from a randomly selected subsample of 25 submissions. The extracted data from the human annotators were compared to the LLM outputs using an LLM-as-judge evaluation method [22]. Additionally, inter-annotator agreement was calculated to establish a human accuracy benchmark.

We evaluated extraction agreement by comparing each annotator’s structured output with the consensus of the other annotators and with the LLM output, using an LLM-as-a-judge rubric to score semantic similarity of extracted fields. LLM-as-a-judge evaluation has been validated for agreement with human preferences in open-ended generation and comparative evaluation settings [23]. In our setting, the LLM’s agreement with the human-consensus benchmark was comparable to inter-annotator agreement and exceeded the agreement of two individual annotators with the benchmark.

The three human annotators were senior medical students with an interest in neurodevelopmental health. Their training was overseen by a senior clinician-researcher with expertise in ADHD care and a strong track record in qualitative health research. The annotators participated in structured training sessions, which included a guided review of sample submissions, discussion of annotation criteria, and iterative feedback on their extraction accuracy. Ongoing support and quality assurance were provided throughout the project to ensure consistent application of the coding schema and maintain reliability.

### 2.4. Data Categorisation

Of the 505 submissions, 25 were used in the AI validation process and subsequently excluded, resulting in a total of 480 submissions.

To enable thematic analysis, all extracted responses were embedded using AI and similar responses were grouped into distinct categories through agglomerative clustering. A second LLM step labelled each cluster to aid interpretability.

The AI-generated categories were reviewed and further grouped by the human annotators into themes. Where a category was relevant to multiple themes, it was assigned to the most pertinent by consensus among the human annotators.

### 2.5. Data Analysis

Descriptive statistics were generated to summarise respondent demographics and thematic frequencies across all submissions. Themes were ranked by frequency within each category (current problems, services wanted, solutions suggested) and graphically visualised as the percentage of respondents providing data for each theme.

## 3. Findings/Results

### 3.1. Demographics

All 480 submissions reported some demographic data. Most authors were female (346 out of 402, 86%). Of the 478 submissions that gave the information, 384 (80%) had ADHD themselves, 147 (31%) disclosed a son, 112 (23%) a daughter, 17 (3.6%) a spouse, and 13 (2.7%) a sibling with ADHD. Of the 315 submissions (65%) disclosing the information, the largest number (45%) came from NSW (Table 1). Age, life stage, and whether responses related primarily to paediatric or adult services were inconsistently reported and could not be summarised reliably.

### 3.2. Current Problems

**Figure 2:**
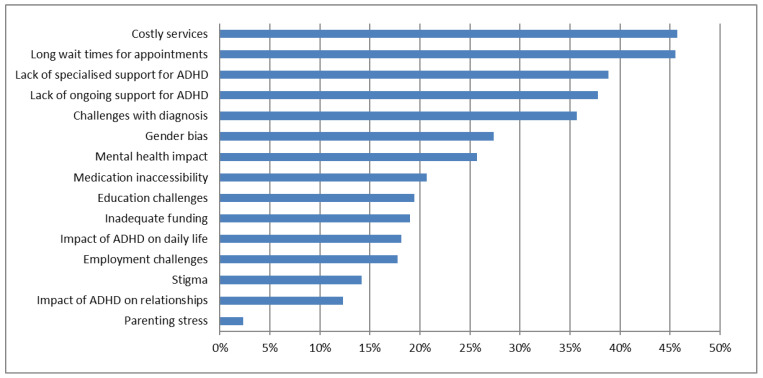
‘Current problems’. AI-extracted ‘Current problems’ have been grouped together into themes. The chart gives the percentage of submissions describing problems for each theme. It is organised in descending order, from highest to lowest frequency.

The major themes were costly services and long wait times, each mentioned in 46% of submissions (Figure 2) (Table 2). Lack of specialised support was mentioned in 39%. This referred to a shortage of specialists and a lack of specialised understanding of ADHD among healthcare professionals. Challenges in diagnosis (36%) included delays due to misdiagnosis and dismissive attitudes of healthcare professionals, and late diagnosis in adulthood. Gender bias, mentioned in 27%, also had implications for diagnosis and treatment. Lack of ongoing support for ADHD was a problem for 38%. Educational and employment challenges (18% and 19%, respectively) are related to inadequate support and lack of understanding and accommodation for ADHD. The submissions illustrated the interactions of the various problems, with undiagnosed and untreated ADHD leading to poor academic performance, underemployment, and job insecurity, thereby reducing earning capacity and impacting the ability to pay for private services.

### 3.3. Services Wanted

**Figure 3:**
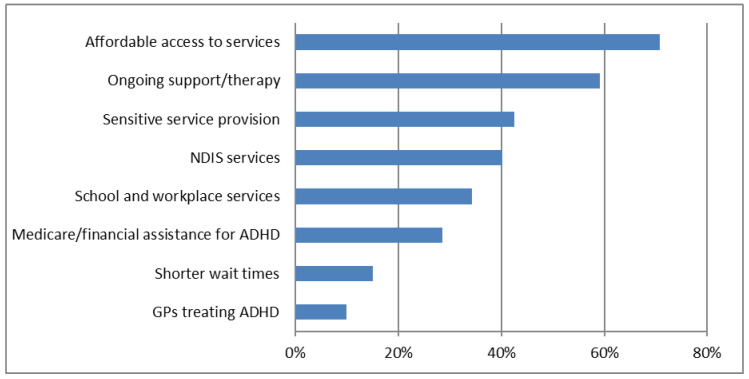
‘Services wanted’. AI-extracted ‘Services wanted’ have been grouped together into themes. The chart gives the percentage of submissions describing problems in each theme. It is organised in descending order, from highest to lowest frequency.

All 480 submissions identified service improvements to better support people with ADHD. The most frequent theme was for affordable access to services, mentioned by 71% (Figure 3) (Table 3). Accessibility also referred to the range of ADHD-specific services available, which 59% mentioned could be improved with post-diagnostic support, life coaching for adults and families, and mental health support. Sensitive service provision (43%) included a desire for services tailored to girls and women, and gender-diverse populations. Many submissions (34%) emphasised the need for educational and workplace support, listing ADHD screening in schools and workplace accommodations for people with ADHD. Recognition of ADHD for NDIS support was a priority for 40%, with 29% wanting Medicare rebates for ADHD diagnostic and mental health services, or some other financial assistance for ADHD-related costs. Ten percent wanted GPs to be able to prescribe ADHD medication.

### 3.4. Solutions Suggested

**Figure 4:**
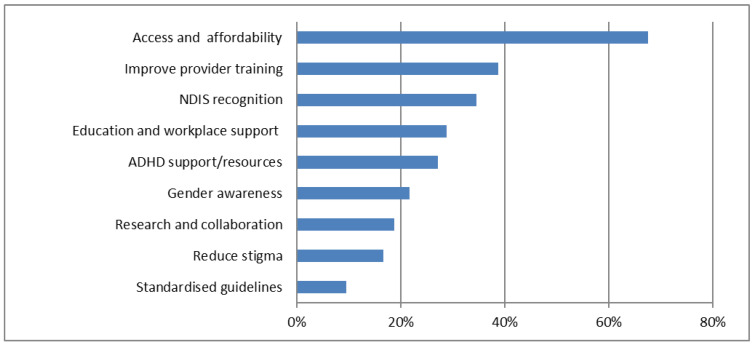
‘Solutions suggested’. AI-extracted ‘Solutions suggested’ have been grouped together into themes. The chart gives the percentage of submissions describing problems in each theme. It is organised in descending order, from highest to lowest frequency.

Solutions to enhance ADHD management were suggested in 478 submissions (>99%). The core message focused on access and affordability, as mentioned by 68% (Figure 4) (Table 4). This included a range of suggestions, including Medicare funding for psychology and psychiatry services, expanding the scope of GPs to prescribe ADHD medications, and expanding coverage by the Pharmaceutical Benefits Scheme (PBS) to ensure equitable access to medication that was not influenced by age of diagnosis. NDIS recognition for ADHD was mentioned by 35%. Improved provider training was a priority for 39%. This included GPs, mental health professionals, and teachers. Support within educational and employment settings was mentioned by 29%, encompassing solutions such as mental health and occupational therapy support for students, as well as neurodiversity support in schools and workplaces. Suggestions for enhancing the availability of ADHD-specific support resources (27%) included a centralised information website and support programmes for individuals with late diagnoses. Submissions supported increased research funding (19%), incorporating neuro-affirming approaches and lived experience. Ten percent supported the development of standardised guidelines for diagnosis and treatment.

## 4. Discussion

### 4.1. Principal Findings

The Senate Inquiry offered a unique opportunity to examine a large repository of lived experience narratives from individuals with ADHD and their families. By applying AI-assisted data extraction and thematic clustering, this study enabled the scalable and structured analysis of these submissions, highlighting key barriers, unmet needs, and proposed solutions. Most submitters described their own ADHD (80%), while a substantial minority also described caring for a son or daughter with ADHD. Respondents described significant systemic barriers to timely diagnosis and treatment, including high out-of-pocket costs, prolonged wait times, and fragmented continuity of care. These challenges were compounded by the daily impacts of living with undiagnosed or unsupported ADHD, affecting education, employment, and family life. The most frequently requested services directly mirrored the challenges reported: improved affordability, timely access to diagnosis and therapy, and ongoing support across the lifespan. Respondents also highlighted the need for gender-sensitive and inclusive service models and greater financial support for ADHD-related healthcare costs. While access and affordability were central concerns, respondents also consistently called for systemic improvements, including enhanced training for healthcare professionals, educators, and employers, to better recognise and support people with ADHD across a range of settings.

### 4.2. Strengths and Limitations

The large sample size is a major strength, however, the dataset represents self-selection and is therefore subject to participation bias. Respondents needed to know about the inquiry and to have the motivation and capacity to give a written submission. Therefore people of non-English backgrounds, lower literacy, or greater ADHD-related impairment may be under-represented. The geographic and demographic reporting was incomplete. From the demographic information provided, the submissions were predominantly from women who themselves had ADHD, with nearly half residing in NSW. Men were under-represented in the submissions (only 14% of respondents). The substantial gender skew in participation may reflect that women are more willing to make written submissions. The high rate of submissions from women with ADHD may reflect their experience of gender-related barriers to the diagnosis of ADHD in women. It is possible that men might respond more easily to other methods such as audio submissions.

A key strength of this study lies in its use of validated AI methods to extract and synthesise data at scale, enabling efficient thematic analysis of over 480 submissions. Validation against three blinded human annotators demonstrated the LLM’s consistency and reliability. Final thematic classification remained under human oversight, preserving interpretative accuracy and accountability. The large sample size supports the validity of the findings. However, a different group of human annotators may have interpreted and grouped the categories differently, potentially leading to variability in data synthesis and presentation.

However, AI validation was based on a small subsample (25 of 505 submissions), which may limit the robustness of model performance assessment. Future work should expand the validation sample and consider iterative human-in-the-loop refinement to further enhance reliability.

A key limitation is that the submissions were prompted by the published terms of reference, which would have biased the responses substantially. Blinding the human annotators to the terms of reference reduces the risk of coders forcing categories to match the inquiry structure, but it does not remove the primary effect on participants’ responses. The numbering of the terms of reference, which might have reflected prioritisation by the organisers, may have introduced further bias by projecting this prioritisation onto the respondents. The higher frequency of issues aligned with earlier terms of reference may partly reflect response fatigue, where respondents provide more detailed content early in a submission and address later prompts less completely. This may attenuate frequencies for later-listed topics and should be considered when interpreting prevalence-ranked outputs. By choosing a different categorisation (problems experienced, services wanted, solutions suggested) and reporting the data themes in order of frequency, we have tried to elucidate the order of priorities of the respondents. It is noteworthy that the major emphasis that the respondents gave to the cost of accessing ADHD services was unprompted by the terms of reference, as was the notion of treatment by GPs.

Because the terms of reference emphasised assessment, treatment access, and formal service structures, submissions may preferentially emphasise ‘medicalised’ solutions (diagnosis pathways, prescribing, specialist availability). By contrast, everyday supports, such as work and school-based accommodations, family supports and neurodiversity-informed service models may be under-represented.

Demographic data on the respondents were not requested in the terms of reference. While some could be inferred from the submissions, the data are incomplete, preventing sub-analysis by different demographic groups.

### 4.3. Comparison with the Senate Inquiry Report

This study analyses the written submissions to the Senate Inquiry by people with lived experience of ADHD. It therefore uses much of the data included in the official report by the Community Affairs Reference Committee [13]. The official report also included evidence from 79 witnesses at three public hearings, as well as submissions from professionals and community support organisations. The official report spans 284 pages, many of which are devoted to direct quotes that provide specific details about people’s experiences. By contrast, in the present study, only information relating to the three key questions was extracted. In this process, the richness of the individual narratives has been lost, but the analysis has been able to quantify by frequency the main problems experienced, services wanted, and solutions suggested. The conclusion of the official report states: ‘Overwhelmingly, people with ADHD want more accessible support to help them thrive and reach their best potential: in their relationships, in their studies and in their work.’ The present study complements this message, making it more powerful by providing the numeric data.

We restricted analysis to written submissions from individuals from families with lived experience, excluding organisational and professional submissions. This improves specificity to consumer priorities but may omit professional themes and system-level considerations more commonly raised by providers, such as research funding, guideline implementation, and workforce modelling.

Unlike the Senate Inquiry’s high-level thematic summary, our LLM-assisted analysis offers a novel, data-driven perspective by identifying and quantifying subthemes that received less emphasis in the official report. This includes issues such as NDIS eligibility, transitional care gaps during adolescence, and the specific needs of gender-diverse individuals with ADHD. By providing prevalence-ranked themes and highlighting service gaps, our approach complements and deepens the understanding of barriers and solutions in ADHD care.

### 4.4. Interpretation and Implications

Findings from this study reveal a critical disconnect between clinical guidelines, current practice, and the realities of individuals navigating ADHD care. Access to diagnosis and treatment appears limited by systemic barriers, including long wait times for specialists, high costs, and state-based regulations. Collectively, these issues result in a fragmented system that limits access to necessary care, thereby exacerbating symptoms and overall distress. Although a range of solutions were proposed to address these issues, a frequently advocated solution was to expand the scope of GPs to diagnose and treat ADHD. This has been partially addressed by recent reforms in NSW [24]. Under the new policy, GPs with accredited training are able to provide ongoing prescriptions for patients with stable doses, with a smaller number enabled to ’diagnose and initiate medications where appropriate.’ However, in most cases, this limits GPs to maintenance prescribing once stability is achieved. Moreover, inconsistencies persist between states. Currently, Queensland alone permits GPs to initiate stimulant medication for children with ADHD aged 4–17 years and has recently extended this to include to adults [25], while Western Australia has announced plans to allow trained GPs to diagnose and treat ADHD but only from age 10 [26]. Therefore, despite the government’s support for one key recommendation to ‘expedite the development of uniform prescribing rules to ensure consistency between state and territory jurisdictions’ [16], nationwide discrepancies persist.

Inconsistencies in ADHD diagnosis and management are further perpetuated by age at diagnosis and gender bias. Submissions frequently reported disparities encountered for those first accessing care as adults, including higher medication costs and limited support services. These experiences reveal substantial gaps in support after childhood. These affect individuals transitioning into adult care or receiving a diagnosis in adulthood.

Submissions further called for more gender-specific research, particularly regarding female presentations. This was strongly advocated, due to continual barriers reported by females in accessing timely ADHD diagnosis and management. Studies corroborate these concerns, reporting a younger age of diagnosis and treatment initiation in males compared to females [27,28]. A contributing factor that may account for this delay is that the manifestation of ADHD symptoms in females may be more subtle, causing them to be overlooked by clinicians [28,29,30]. This reflects a common belief among submissions, with many attributing their delayed diagnosis to a general lack of provider knowledge regarding ADHD in females.

Another notable finding is the misalignment between community perspectives and current evidence regarding ADHD diagnosis and management. This highlights the need for clear communication and broader education around ADHD. For example, many parents supported routine school-based screening for early detection of ADHD. However, this is not recommended in the Australian clinical practice guidelines for ADHD [31], as there is currently no single appropriate screening tool [32]. A second example relates to the NDIS, with submissions urging for ADHD to be recognised as a primary diagnosis to qualify for NDIS support. Currently, NDIS support for ADHD is only granted in association with significant functional impairment or another NDIS recognised primary disability. This broadly accords to the Australian clinical practice guidelines, which emphasise that eligibility should be determined by individual needs rather than diagnosis alone [31]. While this appears logical, as of March 2023, only 40 adults with ADHD as their primary diagnosis were receiving NDIS support [13]. This suggests that the NDIS may systematically minimise the impact of ADHD on daily functioning.

The challenges identified in this study are closely interrelated, often compounding one another. For instance, delays in diagnosis lead to academic and occupational setbacks, which then restrict financial access to care, perpetuating a cycle of disadvantage. Addressing these issues will require not only targeted service improvements but also coordinated, cross-sector reform to build an ADHD care system that is equitable, accessible, and informed by lived experience. As reforms such as expanded GP prescribing are implemented, robust evaluation will be critical to ensure alignment with community needs. Evaluation should include the quality, capacity, and cost-effectiveness of the care, to the satisfaction of the ADHD community. Evaluation will therefore require a major emphasis on consumer feedback. The findings of this study will be useful for assessing and informing future reform.

To date the government response to the Senate Inquiry into ADHD services has been disappointing compared to the Senate Select Committee’s inquiry into and reporting on the services, support, and life outcomes for autistic people [15]. A key recommendation of that Committee’s report, delivered in March 2025, was to develop both a National Autism Strategy and a National Roadmap to Improve the Health and Mental Health of Autistic People [14]. This was supported by AUD 42.2 million in the 2025–2026 budget [33]. This was despite the inquiry into ADHD services having many more submissions (701 compared to only 168) [13,15].

## 5. Conclusions

This study used AI to analyse Senate Inquiry submissions from people with lived experience of ADHD. The most frequently reported barriers were long wait times, affordability, and fragmented continuity of ADHD care. Respondents prioritised affordable, accessible multidisciplinary services, clearer care pathways for people of all ages, broader GP prescribing powers, and better provider training. These results support the need for nationally consistent policies for ADHD care in general and specifically for prescribing. The use of AI to generate frequency-ranked mapping complements the narrative analysis of the Senate Inquiry report by quantifying the most dominant barriers and favoured solutions. This provides guidance to policy makers in designing solutions that can be audited and evaluated according to the priorities of respondents with lived experience of ADHD and can add numerical weight to the more nuanced narrative analysis of the official report [13].

## Figures and Tables

**Table 1:** AI-detected population characteristics from individual submissions.

Characteristic	N *	Percentage (%)
Gender (*n* = 402)		
Female	346	86
Male	56	14
State (*n* = 315)		
ACT	34	11
NSW	143	45
NT	2	1
QLD	13	4
SA	11	3
TAS	12	4
VIC	65	21
WA	35	11

* Based on the number of submissions that included demographic data.

**Table 2:** Current problems.

Theme	AI-Generated Categories
Costly services	Access and Affordability of ADHD Coaching and Support Services
	Financial Barriers to Accessing Therapeutic Services
	Financial Burden of Medical Assessments and Treatments
	Financial Burden of Psychiatric Appointments and Medications
	High Financial Costs of ADHD Diagnosis and Treatment
	High Costs of ADHD Assessments and Diagnosis
	High Out-of-Pocket Costs for Assessments and Therapies
Long wait times for ADHD Appointments	Long wait times for ADHD Appointments
	Long Wait Times for Specialist Appointments
	Appointment Wait Times
	Long Wait Times for Psychiatrist Appointments
	Long Wait Times for Assessments and Treatment
	ADHD Assessment and Treatment Wait Times
	Long Wait Times for Diagnosis and Treatment
	Long Wait Times for Pediatric and Child Psychiatric Services
	Difficulty Accessing Timely ADHD Assessments and Support Services
Lack of specialised support for ADHD	ADHD Specialist Shortage and Access Issues
	Lack of ADHD Awareness and Understanding Among Healthcare Professionals
	Lack of Support and Understanding from Healthcare Professionals
	Limited Availability of Qualified ADHD Specialists in Australia
Lack of ongoing support for ADHD	Barriers to Accessing Affordable and Appropriate Support Services
	Post-Assessment Support Challenges
	Post-Diagnosis Support Challenges
	Inaccessibility of Healthcare and Support Services for Adults with ADHD
	Limited Access to Mental Health Services
Challenges with diagnosis	Challenges in ADHD Diagnosis and Support for Children
	Misdiagnosis of Mental Health Disorders
	Late ADHD Diagnosis in Adulthood
	Dismissive Attitudes of Healthcare Professionals Towards ADHD Diagnosis
	Barriers and Challenges in Obtaining an Adult ADHD Diagnosis
	Lengthy and Complicated ADHD Diagnosis Process
	Lengthy and Complex Diagnostic Process Issues
	Inadequate Access to ADHD Diagnosis
Gender bias	ADHD Awareness and Understanding in Women by Healthcare Providers
	Gender Bias in ADHD Diagnosis and Treatment
	Gender Bias in Medical Diagnosis and Treatment
Mental health impact	Low Self-Esteem and Feelings of Inadequacy
	Daughter’s Emotional and Social Challenges Due to ADHD and Anxiety
	Emotional Regulation and Dysregulation
	Mental Health Challenges Related to ADHD and Emotional Struggles
	Mental Health Challenges and Disorders
Medication inaccessibility	Limitations and Challenges of Medication-Based Treatments
	Strict Medication Access Regulations
	Access Challenges to ADHD Medication Due to Regulations
	Barriers to Affordable Medication Access
Education challenges	Academic and Professional Performance Inconsistencies
	School Refusal and Child Anxiety Impact on Academic Performance
	Inadequate Support for Neurodivergent Students in Higher Education
	Inadequate School Support for Students with ADHD
	Inadequate Communication and Support for Children’s Educational Needs
	Teacher Training on ADHD and Neurodiversity
Inadequate funding	Recognition of ADHD in NDIS
	Insufficient Funding for ADHD Research and Services
Impact of ADHD on daily life	Difficulty with Concentration and Task Management
	Time Management and Organizational Challenges
	Executive Dysfunction and Daily Life Challenges
	Financial Instability and Risk of Homelessness
	ADHD and Financial Impulsivity Challenges
Employment challenges	ADHD and Employment Challenges
	Job Insecurity Due to Inability to Perform
	Work-Related Burnout and Mental Health Challenges
	Workplace Discrimination and Lack of Accommodations for Individuals with ADHD
Stigma	Stigma and Misunderstandings Surrounding ADHD
Impact of ADHD on relationships	Family Relationship Challenges and Parenting Strains
	Negative Impact of ADHD on Personal and Work Relationships
	Challenges in Maintaining Social Relationships
Parenting Stress	Parenting Stress and ADHD

**Table 3:** Services wanted.

Theme	AI-Generated Categories
Affordable access to services	Affordable and Streamlined Diagnostic and Treatment Processes
	Improved Access to ADHD Diagnosis and Support Services
	Affordable and Accessible Mental Health Services
	Access to Affordable Medication and Treatment Options
	Affordable Access to ADHD Medications
	Access to ADHD Medications
	ADHD Medications Coverage under the PBS
	Access to Affordable ADHD Coaching
	Access to Mental Health Professionals
Ongoing support/therapy	Post-Diagnosis Support Services
	Mental Health Support and Counseling
	ADHD Mental Health Support and Resources
	Occupational Therapy
	Support Services for Neurodivergent Individuals
	ADHD Support Groups
	ADHD Support Services for Adults and Families
	ADHD Management and Resources
	ADHD Coaching and Management Strategies
	Increased Availability of ADHD Trained Professionals
Sensitive Service Provision	Gender Bias and Diversity in ADHD Research and Treatment
	ADHD Training for Professionals Focused on Women
	ADHD Awareness and Education in Women and Girls
	ADHD Awareness and Sensitivity Training for GPs
	Comprehensive ADHD Assessment and Diagnosis
	Improved Training and Education for Healthcare Professionals on ADHD
	ADHD Stigma Reduction Campaigns
NDIS services	ADHD Recognition and Government Support
	Recognition of ADHD under NDIS
	Recognition of ADHD in the NDIS
	NDIS Support and Funding for ADHD
School and workplace services	ADHD Screening in School Populations
	Workplace Accommodations for Individuals with ADHD
	Educational Support for Children with ADHD in Schools
	Teacher Training on ADHD Management in Schools
	ADHD and Neurodiversity Training for Educators
	Increased Awareness and Education about ADHD in Schools and Workplaces
Medicare/Financial assistance	Medicare and Subsidized Psychiatric Services for ADHD Diagnosis
	Medicare Rebate for Mental Health Services
	Medicare Rebates for ADHD Services and Assessments
	ADHD Diagnosis and Treatment Funding
	ADHD Support and Government Funding
	Increased Government Funding for ADHD Research
	Financial Assistance for Healthcare Services
	Financial Assistance for ADHD-related Costs
Shorter service wait times	Shorter Wait Times for Assessments and Therapy Services
	ADHD Diagnosis and Assessment
	Timely Access to ADHD Assessment and Diagnosis
GPs treating ADHD	GPs Prescribing ADHD Medication

**Table 4:** Solutions suggested.

Theme	AI-Generated Category
Access and affordability	Increase in Medicare-Funded Psychology Sessions
	Medicare-subsidized Access to Psychiatrists for ADHD Diagnosis
	Medicare Coverage for ADHD Diagnosis and Treatment
	Medicare Rebates for ADHD and Psychiatric Services
	Allowing GPs to Prescribe ADHD Medications
	Standardizing ADHD Medication Prescribing and Access Guidelines
	Equitable Pricing for ADHD Medication Across Demographics
	Expanding ADHD Medication Coverage under the Pharmaceutical Benefits Scheme (PBS)
	Reducing Costs in ADHD Diagnosis and Treatment
	ADHD Financial Assistance and Subsidies
	Improving Accessibility and Affordability of ADHD Assessments and Treatments
	Improving Access to Mental Health Services for ADHD
	Access to Affordable Mental Health Services
	ADHD Assessment and Support Funding
	Increasing Funding for ADHD Diagnosis and Treatment Professionals
	Funding and Access to ADHD Services
	Reducing Wait Times for ADHD Diagnosis and Treatment
	Reducing Wait Times for Healthcare Appointments and Services
Improve provider training	Increasing Sensitivity and ADHD Awareness Training for GPs to Reduce Bias
	ADHD Training for GPs and Mental Health Professionals
	ADHD Training and Support for Teachers
	Early Screening and Assessment for At-Risk Populations and Children with ADHD and Autism
	Increasing ADHD Diagnosis and Treatment Capacity
	Holistic Treatment Approaches for ADHD
NDIS recognition	Expanding NDIS Eligibility and Support for Adults with ADHD
	Advocacy for ADHD Recognition under NDIS
	Inclusion of ADHD in NDIS Eligibility
	ADHD Recognition as a Primary Disability by NDIS
	ADHD as a Primary Disability under NDIS
	Recognizing ADHD as a Primary Disability for NDIS Support
	ADHD Recognition as a Disability
Education and workplace support	Support Strategies for ADHD Students in Educational Institutions
	Supporting Neurodiversity in Education and Workplace
	Workplace Support and Education for ADHD
	Increasing Access to Mental Health and Occupational Support in Schools
ADHD support and resources	Improving Accessibility and Support Services for ADHD
	Centralised ADHD Resource Website
	ADHD Advocacy and Accessible Resources
	ADHD Support Groups and Resources
	Support Programs for Late-Diagnosed Individuals
	ADHD Coaching Accessibility and Accreditation
Gender awareness	ADHD Research and Gender-Specific Considerations for Women and Girls
	ADHD Awareness and Training for Women and Gender-Diverse Individuals
Research and collaboration	ADHD Research in Diverse Populations and Neuroaffirming Approaches
	Increased Funding for ADHD Research
	Incorporating Lived Experiences in ADHD Service Development and Research
	Improved Communication and Collaboration Across Services and Sectors
Reduce stigma	Community Awareness and Education on ADHD
	Increasing Awareness and Reducing Stigma Around ADHD
	ADHD Awareness and Stigma Reduction Campaigns
Standardised guidelines	Standardizing National Guidelines for ADHD Diagnosis and Treatment
	Streamlined ADHD Diagnosis and Treatment Processes

## Data Availability

The data are freely available at https://www.aph.gov.au/Parliamentary_Business/Committees/Senate/Community_Affairs/ADHD/Report, accessed on 1 December 2024 [13].
